# Hereditary elliptocytosis in a child with an autosomal recessive *SPTA1* mutation: a case report from Saudi Arabia

**DOI:** 10.25122/jml-2025-0038

**Published:** 2025-08

**Authors:** Fahad Alamr

**Affiliations:** 1 Pediatric Department, Faculty of Medicine, Al-Baha University, Al-Baha, Saudi Arabia

**Keywords:** hereditary elliptocytosis, *SPTA1* gene mutation, autosomal recessive inheritance, genetic counseling, hemolytic anemia, molecular genetics

## Abstract

Hereditary elliptocytosis (HE) is a genetic red blood cell disorder characterized by elliptical-shaped cells. Clinical manifestations range from asymptomatic cases to severe hemolysis and are influenced by underlying gene mutations, including those in spectrin alpha, erythrocytic 1 (*SPTA1*). This case report examines whether zygosity of the mutation correlates with clinical severity. The case involved a comprehensive diagnostic approach including complete blood count, peripheral blood smear, sodium dodecyl sulfate-polyacrylamide gel electrophoresis, and whole exome sequencing using the CentoXome MOx Solo platform (CENTOGENE, Rostock, Germany). The analysis focused on coding regions and adjacent intronic nucleotides of genes associated with known phenotypes, specifically looking at the correlation between mutation zygosity and clinical severity. The patient, a 10-year-old Saudi male, presented with mild normocytic normochromic anemia and signs of extravascular hemolysis. A significant percentage of elliptocytes was noted on the blood smear. Genetic analysis revealed a homozygous likely pathogenic variant in the *SPTA1* gene, specifically NM_003126.2.779T>C (p. Leu260Pro). This mutation is associated with autosomal recessive HE, suggesting a possible correlation between homozygosity and a more pronounced clinical presentation. Identifying a rare homozygous mutation in the *SPTA1* gene confirms the diagnosis of autosomal recessive HE in a Saudi Arabian pediatric patient. It suggests a correlation between homozygosity and the severity of anemia and hemolysis.

## INTRODUCTION

Hereditary elliptocytosis (HE) is a genetic red blood cell (RBC) disorder characterized by elliptically shaped erythrocytes resulting from defects in cytoskeletal proteins of the RBC membrane [1,2]. Abnormal cell morphologies, including ovalocytes, rod-shaped cells, and RBC fragments, are typically observed on peripheral blood smears. The condition is primarily caused by pathogenic variants in genes encoding key cytoskeletal proteins such as *SPTA1* (α-spectrin), *SPTB* (β-spectrin), *EPB41* (protein 4.1), and *SLC4A1* (band 3), and less commonly *GYPC* (glycophorin C) [1]. The spleen plays a significant role in HE, removing these abnormal elliptocytes and causing hemolytic anemia. First described by Dresbach in 1904 and later established as hereditary by Hunter, HE encompasses several subtypes, including common HE, hereditary pyropoikilocytosis (HPP), spherocytic elliptocytosis (SE), and Southeast Asian ovalocytosis (SAO), each defined by distinct RBC morphology and varying degrees of hemolysis [2]. While most individuals with HE are asymptomatic and require no treatment, symptomatic cases may be managed with blood transfusions or splenectomy.

The global prevalence of HE is estimated at 1 in 2,000–4,000 individuals, though it is more common in certain African populations, with a prevalence of approximately 1 in 1,100 [3]. It is predominantly inherited in an autosomal dominant pattern and results from gene mutations responsible for RBC membrane or skeletal proteins. These mutations disrupt the horizontal interactions between membrane skeletal proteins and the cell membrane, reducing RBC deformability and impairing membrane function. The most frequently affected genes are *SPTA1* (spectrin alpha, erythrocytic 1; ≈65%), *SPTB* (β-spectrin; ≈30%), and *EPB41* (erythrocyte membrane protein 4.1R; ≈5%) [4]. Clinically, HE presents with a wide range of phenotypes, ranging from asymptomatic to varying degrees of hemolysis, including hemolytic compensatory and hemolytic anemia types. Less than 10% of individuals with HE develop the more severe form, HPP. The distribution of HE correlates with regions where malaria is endemic, suggesting that the disease may have influenced the spread of the genetic mutation responsible for this condition [2]. Certain variants, such as SAO, are particularly common in populations from Malaysia, Papua New Guinea, Indonesia, and the Philippines, with prevalence rates ranging from 5% to 25%. In contrast, SE is more frequently seen in individuals of European descent [3,5]. Hereditary blood disorders are prevalent in many parts of the world, including Saudi Arabia and the broader Middle East. In some areas of Saudi Arabia, over 10% of the population may carry a gene for one of these disorders [6].

Diagnosing HE includes a complete blood count, which often reveals mild normocytic, normochromic anemia. Peripheral blood smear analysis demonstrates a significant proportion of elliptocytes, ranging from 15% to 100% of red blood cells [2,7]. Other abnormal cell morphologies, such as spherocytes and poikilocytes, may also be present. Laboratory evidence of extravascular hemolysis, including reticulocytosis, elevated lactate dehydrogenase (LDH), increased indirect bilirubin, and decreased haptoglobin, is frequently observed. Notably, the proportion of elliptocytes does not directly correlate with the severity of hemolysis, although polychromasia due to reticulocytosis is common [2,7].

Further diagnostic measures include sodium dodecyl sulfate-polyacrylamide gel electrophoresis (SDS-PAGE) to precisely quantify erythrocyte membrane proteins such as protein 4.1 and spectrin. Ektacytometry may also be conducted to assess the mechanical properties of the erythrocyte membrane under stress. For cases presenting with splenomegaly, ultrasound is an efficient diagnostic tool, with CT and MRI available if more detailed imaging is necessary [7].

Overall, the diagnosis of HE relies on a combination of medical history, peripheral smear findings, and confirmatory tests such as SDS-PAGE [7,8]. However, due to the wide variability in clinical presentations, relying solely on these methods can lead to missed diagnoses. The introduction of high-throughput sequencing has significantly improved diagnostic accuracy, enabling characterization of HE at the molecular level. This case report highlights the clinical and genetic significance of a homozygous missense variant, NM_003126.2:c.779T>C (p.Leu260Pro), in the *SPTA1* gene in a Saudi child with HE. It also explores how autosomal recessive inheritance may influence disease severity and management in populations with high rates of consanguinity, such as Saudi Arabia.

## CASE REPORT

A 10-year-old Saudi boy from Al-Baha, Saudi Arabia, presented to our hematology clinic with a lifelong history of anemia, as reported by his father. He experienced prolonged neonatal jaundice during the first month of life but did not receive specific treatment (demographic and clinical data are summarized in Table 1). During the first 6 months of life, he required multiple blood transfusions, though none were needed thereafter. He had previously been diagnosed with mild hereditary spherocytosis at another institution, where he intermittently received folic acid supplementation. Despite his medical history, the patient was performing well academically. His parents are not first-degree relatives, though both originate from the same tribe, and they have five other healthy children. There is no known family history of chronic disease.

**Table 1:** Demographic characteristics, clinical history, and examination findings of the patient

Characteristic	Findings
**Age**	10 years, 2 months
**Sex**	Male
**Place of origin**	Al-Baha, Saudi Arabia
**Residence**	Al-Baha, Saudi Arabia
**Family history**	Unknown
**Consanguinity**	No, but the parents are from the same tribe
**Siblings**	Five healthy siblings
**Vaccination status**	Up to date
**Diet**	Family diet
**Symptoms**	Anemia since birth
**Jaundice after birth**	Prolonged jaundice in the first month (no treatment)
**Blood transfusions**	Multiple transfusions in first six months; none thereafter
**Folic acid treatment**	On and off folic acid supplementation
**School performance**	Good
**Physical examination findings**	Mild pallor, no jaundice, no dysmorphism
**Lymphadenopathy**	None
**Splenomegaly**	None
**Hepatomegaly**	None

## PHYSICAL EXAMINATION AND HEMATOLOGICAL TESTS

Upon physical examination, the patient appeared mildly pale but showed no signs of jaundice or dysmorphism. He was vitally stable, with an appropriate height and weight for his age. No lymphadenopathy, splenomegaly, or hepatomegaly was noted. The laboratory tests, shown in Table 2, demonstrated that his white blood cell count (WBC) was 5.5 x 10^9/L, hemoglobin (Hb) was 11.3 g/dL (which is slightly low for his age), mean corpuscular volume (MCV) was 73 fL, hematocrit (Hct) of 35%, and platelet count was 424 x 10^9/L, all within normal ranges except for the low hemoglobin and MCV values. His iron profile was normal, and his reticulocyte count was elevated at 3.2%, suggesting increased RBC production. His total bilirubin was 20.8 µmol/L, and his direct bilirubin was 5.5 µmol/L, both elevated, indicating mild hemolysis.

**Table 2:** Laboratory findings of the patient

Test	Result	Reference Range
**White Blood Cells (WBC)**	5.5 × 10^9^/L	4.5–13.5 × 10^9^/L
**Hemoglobin (Hb)**	11.3 g/dL	12.5–15.5 g/dL
**Mean Corpuscular Volume (MCV)**	73 fL	77–95 fL
**Hematocrit (Hct)**	35%	36–45%
**Platelets**	424 × 10^9^/L	150–450 × 10^9^/L
**Reticulocytes**	3.20%	0.5–1.5%
**Total Bilirubin**	20.8 µmol/L	1.7–17 µmol/L
**Direct Bilirubin**	5.5 µmol/L	0–5 µmol/L
**Ferritin**	56 ng/mL	15–200 ng/mL
**Serum iron**	97 µg/dL	60–170 µg/dL
**TIBC**	275 µg/dL	250–400 µg/dL
**Transferrin saturation**	28 %	20–50 %
**HbA**	96.4 %	—
**HbA_2_**	2.8 %	<3.5 %
**HbF**	0.8 %	<2 %

**Figure 1:**
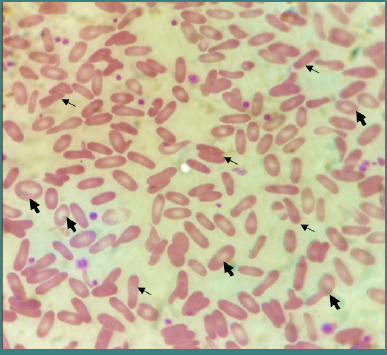
Peripheral blood smear of the patient. Thin arrows (→): elliptocytes, thick arrows (→): anisopoikilocytes

A peripheral blood film (Figure 1) revealed anemia with anisopoikilocytosis and more than 15% elliptical-shaped RBCs, consistent with a diagnosis of HE. The ultrasound examination showed no signs of organomegaly or gallstones. Based on these findings, the patient was maintained on daily folic acid supplementation and underwent whole exome sequencing (WES) for further investigation to confirm the HE diagnosis and identify the pathogenic gene. The WES analysis (Table 3) identified a homozygous missense variant in the *SPTA1* gene, specifically NM_003126.2.779T>C, resulting in a (p. Leu260Pro) amino acid substitution. This variant, associated with rs121918634, is classified as likely pathogenic (Class 2) according to the American College of Medical Genetics and Genomics (ACMG) guidelines. In silico tools, including PolyPhen-2 (probably damaging) and MutationTaster (disease-causing), indicated a high likelihood of pathogenicity. The Align-GVDG score of C65 further suggested that this variant significantly interferes with the protein’s function. The variant is rare, with allele frequencies reported as 0.000014 in gnomAD and 0.00046 in CentoMD. This finding is consistent with an autosomal recessive *SPTA1-*related disease, confirming the HE diagnosis. No other clinically significant variants were identified.

**Table 3:** Genetic variant analysis of the *SPTA1* gene

Sequence Variants	
**Gene**	*SPTA1*
**Variant Coordinates**	NM_003126.2: c.779T>C
**Amino Acid Change**	(p. Leu260Pro)
**SNP Identifier**	rs121918634
**Zygosity**	Homozygous
**In-Silico Parameters**	PolyPhen: Probably damaging Align-GVGD: C65 SIFT: Deleterious MutationTaster: Disease causing Conservation_nt: High Conservation_aa: High
**Allele Frequencies**	gnomAD: 0.000014 ESP: - 1000G: 0.000012 CentoMD: 0.000046
**Type and Classification**	Missense Likely Pathogenic (Class 2)

The patient remained stable, with regular follow-up in the hematology clinic and ongoing folic acid supplementation. Given the genetic findings, targeted testing of the parents was recommended to confirm the variant’s homozygosity and rule out compound heterozygosity. Genetic counseling, including reproductive counseling, was also advised for the family.

## SEQUENCING AND MOLECULAR DIAGNOSIS

The patient’s genetic analysis for HE was performed using the CentoXome MOx Solo platform (CENTOGENE, Rostock). Genomic DNA underwent enzymatic fragmentation followed by enrichment of target regions using DNA capture probes. The enriched regions comprised approximately 41 Mb of the human coding exome, covering >98% of the coding RefSeq (based on the GRCh37/hg19 human genome build) as well as the mitochondrial genome.

The generated library was sequenced on an Illumina platform (Illumina, Inc., San Diego, CA), ensuring at least 20x coverage depth for over 98% of the targeted bases. Using an in-house bioinformatics pipeline, the resulting sequence reads were aligned to the GRCh37/hg19 genome assembly and the revised Cambridge Reference Sequence of the Human Mitochondrial DNA (NC_012920). This pipeline included variant calling, annotation, and comprehensive variant filtering. Variants with a minor allele frequency of less than 1% in the Genome Aggregation (gnomAD) database and disease-causing variants reported in the Human Gene Mutation Database (HGMD), ClinVar, or CentoMD were thoroughly evaluated. The analysis focused on coding exons and flanking +/-10 intronic nucleotides of genes with established gene-phenotype associations, as referenced in the Online Mendelian Inheritance in Man (OMIM).

Various inheritance patterns were considered in conjunction with the patient’s family history and clinical information to assess the pathogenicity and disease causality of identified variants. Variants were categorized into five classes: pathogenic, likely pathogenic, variants of uncertain significance, likely benign, and benign, following the ACMG guidelines for variant classification. Only variants relevant to the patient’s phenotype were reported. Biochemical analysis was performed to supplement the diagnosis of metabolic disorders and optimize the classification of variants in cases where applicable. CENTOGENE adhered to stringent quality control measures, confirming variants with low sequencing quality or ambiguous zygosity through orthogonal methods. This ensured a specificity of over 99.9% for all reported variants. Mitochondrial variants were reported if heteroplasmy levels exceeded 15%. Additionally, copy number variation detection was highly sensitive, achieving more than 95% accuracy for all homozygous/hemizygous deletions, duplications, and mitochondrial deletions involving at least three consecutive exons. Uniparental disomy screening was also conducted using a specific algorithm to assess known clinically relevant chromosomal regions.

## FINDINGS AND OUTCOME

Laboratory evaluation revealed mild microcytic anemia, hemoglobin 11.3 g/dL with an MCV of 73 fL, plus reticulocytosis (3.2 %), indirect hyperbilirubinemia, and more than 15 % elliptocytes on the peripheral blood film. Whole-exome sequencing uncovered a homozygous missense change in *SPTA1* (NM_003126.2: c.779T>C; p. Leu260Pro; rs121918634) that meets ACMG criteria for a 'likely pathogenic' variant, with no other disease-related mutations identified. Clinically, the child remained stable, needed only daily folic-acid supplementation, and did not require transfusions since infancy. Ongoing hematology follow-up was in place, and targeted testing for parents, along with genetic and reproductive counselling, was recommended.

## DISCUSSION

Hereditary erythrocyte disorders are prevalent in many parts of the world, including the Middle East. In some regions of the Middle East, over 10% of the population carry genes associated with hereditary blood disorders [9]. Among them, HE is typically inherited as an autosomal dominant disorder with a wide range of clinical presentations, from asymptomatic carriers to individuals experiencing severe, potentially life-threatening hemolysis [2]. Cases of neonatal jaundice, hemolytic anemia, and even hydrops fetalis have been documented. While mutations in several genes encoding the erythrocyte membrane skeleton components can result in HE, mutations in the *SPTA1* gene are the most common. Our patient presented with anemia, anisopoikilocytosis, and more than 15% elliptical RBCs on peripheral blood smear, consistent with HE.

The *SPTA1* gene, located on chromosome 1q22–q23, spans approximately 80,000 nucleotides and comprises 52 exons. It encodes the α-spectrin protein, a critical subunit of the spectrin heterodimer, which is essential for the erythrocyte membrane skeleton and protects RBCs from shear stress during circulation [10]. Mutations in *SPTA1* that affect α-spectrin function increase RBC fragility, leading to hemolysis. Most pathogenic variants occur near spectrin’s self-association site, where they substitute highly conserved amino acids and disrupt spectrin–spectrin interactions [8,10]. Although a few rare mutations have been described outside this region, no pathogenic variants have yet been reported in the final exon (exon 52) of *SPTA1*.

In this case report, WES identified a homozygous missense variant in the *SPTA1* gene (NM_003126.2.779T>C), resulting in a (p. Leu260Pro) amino acid substitution. This variant is associated with rs121918634 and is classified as likely pathogenic (Class 2) according to the ACMG guidelines. In silico predictive tools such as PolyPhen-2 ('probably damaging') and MutationTaster ('disease-causing') support its pathogenicity, and the Align-GVGD score of C65 indicates a significant impact on protein function. The rarity of this variant in population databases (allele frequency of 0.000014 in gnomAD and 0.00046 in CentoMD) further underscores its clinical significance. Mutations in *SPTA1*, which encodes the α-spectrin protein, are among the most common causes of HE. These mutations often occur near the spectrin’s self-association site, which is important for spectrin dimer and tetramer formation, affecting the RBC membrane’s mechanical stability [10]. However, mutations in other regions of the gene have also been reported. Xi *et al*. described a heterozygous deletion in exon 52 of SPTA1 (c.7220_7221del, p. Tyr2407*) in a Chinese family, leading to a truncated α-spectrin protein and an HE phenotype [10]. Similarly, Shih *et al*. reported a novel heterozygous mutation c.86A>C (p. Gln29Pro) in exon 2 of *SPTA1* in a Taiwanese patient with HE, highlighting that mutations throughout the gene can contribute to the disease [11].

Our case contributes to the literature by identifying a homozygous missense mutation in exon 4 of the *SPTA1* gene, further highlighting the genetic heterogeneity of hereditary elliptocytosis. The (p. Leu260Pro) substitution is located outside the common self-association site but still results in the HE phenotype, suggesting that mutations in various regions of *SPTA1* can impact α-spectrin function and lead to disease manifestation. The patient’s clinical presentation was mild, with slight anemia and an elevated reticulocyte count, indicating compensatory erythropoiesis. Physical examination showed mild pallor without jaundice, splenomegaly, or hepatomegaly. Similar mild phenotypes have been reported in other studies. Al Khairy *et al*. investigated HE in Saudi Arabia and found that 64% of patients displayed detectable protein abnormalities, while 36% were normal, indicating variability in clinical and laboratory findings [12]. This variability underscores the importance of considering HE in patients with unexplained anemia and elliptocytosis, even in the absence of severe symptoms. Interestingly, HPP, a severe form of HE, has been reported in Saudi children by Mallouh and colleagues. They described HPP as an autosomal recessive condition occurring in families with unaffected parents and two affected offspring, illustrating that recessive inheritance can produce more severe phenotypes [13]. In our case, the presence of a homozygous *SPTA1* mutation may explain the occurrence of HE in the absence of a significant family history, further supporting an autosomal recessive mode of inheritance.

Nair *et al*. reported a patient with hemolytic anemia carrying mutations in both *SPTA1* (c.6531-12C>T) and *SLC4A1* (Pro868Leu). While these mutations alone were not pathogenic, their coexistence could cause hemolysis, highlighting the potential for compound heterozygosity or multiple gene interactions contributing to the clinical phenotype [14]. This suggests that genetic interactions can modulate disease severity and presentation. The management of HE is generally supportive. Our patient was maintained on daily folic acid supplementation to support erythropoiesis. He remained clinically stable without signs of significant hemolysis, consistent with other reports of mild HE phenotypes [11]. Splenectomy is reserved for severe cases with significant hemolysis or anemia. Genetic counseling is essential due to the hereditary nature of the disorder, especially in autosomal recessive cases like ours. Interestingly, Alharbi *et al*. reported a novel pathogenic mutation in a child with hereditary spherocytosis associated with von Willebrand disease in Saudi Arabia. They noted that while there was no definitive link between the two hematological disorders, the high prevalence of consanguinity in the region may lead to the co-occurrence of multiple hereditary diseases [15]. Although our patient did not present additional hematological conditions, this highlights the importance of comprehensive genetic evaluation in populations where consanguinity is common to detect potential co-existing disorders.

Our findings emphasize the importance of genetic testing in patients with unexplained hemolytic anemia and elliptocytosis. Identifying specific mutations in the *SPTA1* gene can confirm the diagnosis, inform prognosis, and guide management. Awareness of the genetic variations and clinical spectrum is important for accurate diagnosis and appropriate family counseling. Previous studies have indicated that HE and related disorders might be underdiagnosed or misdiagnosed, particularly in certain populations [10-14]. In the future, it is advised to conduct targeted genetic testing on the parents to ensure accurate genetic assessment and further understanding of the inheritance pattern. This will confirm whether the identified variant’s homozygosity is genuine or a result of compound heterozygosity involving a large deletion. Furthermore, it is recommended that targeted genetic testing be extended to any potentially affected family members to identify carriers and better understand the family’s genetic landscape. Comprehensive genetic counseling should be provided to the family, including discussions on reproductive options, if applicable, to inform future family planning and manage potential genetic risks.

## CONCLUSION

This case report contributes to the understanding of HE and its genetic heterogeneity, particularly in the Middle Eastern population. It demonstrates that mutations across the *SPTA1* gene, including less commonly affected exons, can lead to the disease. Genetic analysis is an essential diagnostic option in confirming the diagnosis, as well as appropriate management and genetic counseling, particularly in atypical presentations. The outcomes suggest the need to consider HE in the differential diagnosis of unexplained anemia and the importance of genetic counseling for affected families. Given the observed autosomal recessive inheritance pattern, genetic counseling is recommended for the family to understand the recurrence risk and make informed reproductive decisions. The early recognition and diagnosis of HE are essential for optimal patient care. Supportive management, including folic acid supplementation and regular monitoring, can help maintain patient stability and quality of life. Our findings highlight the need for increased awareness of the genetic and clinical heterogeneity of HE among healthcare professionals, which can lead to better diagnosis, management, and counseling of affected individuals and their families. The main limitation of this case report is that it describes only one patient, so the genotype–phenotype link seen for the *SPTA1* p. Leu260Pro variant may not apply to the broader population. In addition, functional studies of α-spectrin were not performed, and extended family testing was not undertaken. Therefore, we cannot definitively confirm the pathogenicity of this variant or establish its precise inheritance pattern.

## Data Availability

The data supporting the conclusions of this study are available from the corresponding author upon reasonable request.
